# Self-identified strategies to manage intake of tempting foods: cross-sectional and prospective associations with BMI and snack intake

**DOI:** 10.1017/S1368980024000697

**Published:** 2024-03-20

**Authors:** Jennifer Gatzemeier, Laura L Wilkinson, Menna J Price, Michelle D Lee

**Affiliations:** School of Psychology, Swansea University, SA2 8PP Swansea, UK

**Keywords:** Snack intake, BMI, Tempting food, Strategies, Weight management

## Abstract

**Objectives:**

Individuals often use self-directed strategies to manage intake of tempting foods, but what these strategies are and whether they are effective is not well understood. This study assessed the frequency of use and subjective effectiveness of self-directed strategies in relation to BMI and snack intake.

**Design:**

A cross-sectional and prospective study with three time points (T1: baseline, T2: 3 months and T3: 3 years). At T1, demographics, frequency of use and subjective effectiveness of forty-one identified strategies were assessed. At T2 and T3, current weight was reported, and at T2 frequency of snack intake was also recorded.

**Setting:**

Online study in the UK.

**Participants:**

Data from 368 participants (M_age_ = 34·41 years; M_BMI_ = 25·06 kg/m^2^) were used for analysis at T1, *n* = 170 (46·20 % of the total sample) at T2 and *n* = 51 (13·59 %) at T3.

**Results:**

Two strategy factors were identified via principal axis factoring: (1) diet, exercise, reduction of temptations, and cognitive strategies, and (2) planning, preparation and eating style. For strategy 1, frequency of use, but not subjective effectiveness, was positively related to BMI at T1. Subjective effectiveness predicted an increase in BMI from T1 and T2 to T3. No relationship to snack intake was found. For strategy 2, frequency of use was negatively related to BMI at T1. Neither frequency of use nor subjective effectiveness were related to changes in BMI over time, but subjective effectiveness was negatively correlated with unhealthy snack intake.

**Conclusion:**

Self-directed strategies to reduce the intake of tempting foods are not consistently related to BMI or snack intake.

Body weight is influenced by a range of factors such as genetic predisposition and the environment^(1)^. Weight management approaches will often try to help individuals to manage these factors to reduce and/or maintain their body weight, such as formal cognitive/behavioural weight loss/maintenance intervention^(2)^ or losing/maintaining weight on their own using a variety of cognitive/behavioural self-directed approaches^(3)^. Much is known about the effectiveness of these formal weight loss interventions^(4)^, but less is known about self-directed efforts and how these efforts support the maintenance of changes in eating behaviours. Hartmann-Boyce *et al.* ^(3)^ conducted a systematic review of qualitative studies to investigate cognitive and behavioural strategies for self-directed weight loss. The types of strategy used most often were, for example, restriction or avoidance of specific foods or settings, scheduling of food or physical activity, professional support, and self-experimentation to decide whether to continue a particular approach. Notably, they found that these approaches were not always in line with those recommended within more formal interventions. Weight control registries from USA, Finland and Portugal have found similar approaches to maintain weight loss successfully such as a higher frequency of meals, planning of meals in advance and having healthy snacks^(5–8)^.

Many of the strategies mentioned are also likely to be useful for people who are trying to generally ‘eat healthily’ (i.e. without a specific weight loss or maintenance goal). And related to this is the use of strategies to specifically manage intake of high-caloric, low-nutrient-density tempting foods which are ubiquitous in the ‘Western’ food environment^(9)^. Gatzemeier *et al.* ^(10)^ conducted a qualitative study to examine ways in which individuals manage their intake of discretionary food items day to day. Identified strategies fell into four broad areas: (1) implementation of cognitive strategies, (2) manipulation of the availability of tempting food and drinks, (3) the strategic formation of meals and (4) the use of exercise.

However, much of the research presented has investigated *which* strategies are used rather than how frequently they are used and if they are subjectively effective. This context is likely to be important when formulating advice for people who are trying to undertake self-directed weight loss, weight maintenance or general management of their diet. For example, areas that may warrant particular focus are helpful strategies that are under-used by target populations or unhelpful strategies that are over-used by target populations. In addition, acceptability of approaches has been shown to relate to subjective effectiveness^(11,12)^ and although individuals are likely to try a range of different approaches, once they find strategies which they find acceptable and perceive them as effective, it is likely that they adopt these in the long term^(13,14)^. Additionally, for many strategies that people have mentioned using in the above study to manage intake of tempting foods^(10)^, there lacks any evidence of objective effectiveness that can be drawn on, especially when strategies are used in combination.

Therefore, the current study assesses the frequency of use and subjective effectiveness of strategies used to manage intake of tempting foods and how this relates to BMI. The strategies investigated were taken from previous qualitative work^(10)^. Additionally, we considered the relationship of frequency of use and subjective effectiveness of strategies with proxies for objective effectiveness by assessing change of BMI over time (from the initial survey to follow-ups 3 months and 3 to 4 years later) as well as snack intake at the 3-month follow-up.

The following pre-registered hypotheses were tested: (1) the frequency of use as well as the subjective effectiveness of the strategies will negatively correlate with BMI (The pre-registered hypothesis predicted a difference in frequency of use and subjective effectiveness between BMI categories. This hypothesis was adapted from the pre-registered hypothesis to reflect BMI as continuous variable and to be directional); (2) higher frequency of use and higher subjective effectiveness maintains or reduces the BMI over the 3-month follow-up period (T2); and (3) a higher reported frequency and subjective effectiveness of the strategies at the initial survey will predict a lower self-reported intake of unhealthy snacks at the follow-up (T2). It was also predicted that BMI would decrease or stay stable over the 3- to 4-year follow-up period (T3) if frequency of use and subjective effectiveness are high (assessed in an exploratory analyses).

## Methods

### Participants

Participants for the survey at T1 were recruited UK-wide via social media (e.g. Facebook and Twitter/X), websites designed to recruit participants (e.g. https://www.surveycircle.com/en/) and an internal participant pool for psychology students with the following exclusion criteria: under 18 years old, pregnant or breast-feeding, taking medication or being diagnosed with a condition influencing appetite, having a history of or current diagnosis of an eating disorder, and low proficiency in English. Also participants with a BMI < 18 kg/m^2^ were excluded to minimise the risk of including individuals with a possible eating disorder. This was not advertised as exclusion criteria, as individuals might not know their BMI, but participants were excluded at the stage of data cleaning.

In total, 675 individuals started the survey, with 368 participants’ data available for analysis at T1 (128 individuals did not give consent, 161 did not finish the survey, 9 skipped eligibility questions and 9 reported a BMI < 18 kg/m^2^). Participants who took part in the initial survey (T1) were asked if they would be willing to participate in a follow-up. If they agreed, they were contacted again 3 months (T2) and 3 years (T3) later. Totally, 306 participants indicated their interest in completing the follow-up questionnaire at T1. Of these, 170 participants (46·20 % of the total sample) completed the first follow-up questionnaire (T2) and 51 (13·86 %) the second follow-up (T3) and provided data that were available for analysis (one individual was excluded due to a BMI below 18 kg/m^2^ in the first follow-up).

This exceeded the requirements of our a priori sample size calculation (using G * Power) which indicated a minimum sample size of 199 participants (small-to-medium effect size (0·2), *α* = 0·05, 1-*β* = 0·8, and four covariates). This calculation was based on an ANCOVA approach, as advised by Hayes and Preacher^(15)^ as a reasonable alternative when moderation sample size approaches are not available. This also covers the required sample sizes of 83 and 155 for the other analyses (see supplemental data A for further details).

Participants were entered into a prize raffle for two Amazon e-vouchers of £25 for taking part in the study. The baseline survey (T1) and the first follow-up (T2) are pre-registered at OSF (https://osf.io/4b2ex/ ). The second follow-up (T3) was part of a student project and therefore not pre-registered.

### Materials

All questionnaires were designed and presented using Qualtrics^(16)^.

#### Demographics

Participants demographics were collected including age, gender, gender identity, ethnicity, occupation, living condition, health information (smoking and drinking behaviour), dieting behaviour (currently dieting and how many times they dieted in the last 3 years), if they had bariatric surgery, meal style, physical activity, and weight and height (to calculate BMI and weight suppression which is the difference between the highest weight since current height (excluding pregnancy) and current weight).

#### ‘Frequency’ and ‘Effectiveness’ questionnaire

The questions in this questionnaire are based on focus group discussions about strategies people use to manage intake of tempting foods in their everyday lives^(10)^. We did not give a definition of ‘tempting foods’ as individuals may be tempted by different foods. For each strategy identified (*n* = 41), individuals had to indicate the frequency of use of a given approach (e.g. ‘How often do you cook your meals for a few days or the whole week in advance?’) and if they used it, the subjective effectiveness was also assessed (e.g. ‘How effective is cooking meals in advance in limiting your intake of tempting food?’). Frequency of use was measured on a five-point Likert scale ranging from ‘Never’ (1) to ‘Always’ (5) and subjective effectiveness on a 100-point VAS scale with the end points of ‘Not at all’ and ‘Extremely’. The development of the questionnaire is described in detail elsewhere^(10)^.

#### Self-reported snack intake

Self-reported frequency of intake of twenty-two snack groups, for example, fruits, milk, chips or ice cream, were assessed^(17)^. The scale ranges from ‘Never or less than once a month’ to ‘More than 3 times a day, every day’ on an eight-point scale. A subscale for healthy and unhealthy snack can be created by summation of respective items. The labels ‘healthy’ and ‘unhealthy’ were kept for ease of interpretation and were based on the labels and classification of Brown *et al.* ^(18)^.

### Procedure

The study followed a repeated measures design with an initial survey (T1; 2017/2018), a 3-month (T2) and a 3/4-year follow-up (T3; 2021) survey.

For T1, after indicating consent, participants answered questions about demographics such as age, weight and height to calculate BMI, gender, sociodemographic data and current dieting. Next, the frequency of strategy use and the subjective effectiveness of the strategies used to manage tempting food intake were assessed. This was followed by a battery of psychological and eating behaviour trait measures (for a list of measures, see supplemental data B) which were not used for these analyses. E-mail address was recorded if participants gave consent to take part in a follow-up survey. Participants who did not agree to the follow-up were debriefed and thanked for their participation at the end of the initial questionnaire.

If participants indicated their willingness to take part in the follow-up questionnaire, they received a Qualtrics link 3 months after completing the first questionnaire, with a maximum of three reminders, each 1 month apart. At T2, consent had to be given again and participants had to report their current weight, the adoption of any new strategy undertaken in the last 3 months from a list of the strategies presented, and snack intake before being debriefed. In order to keep the surveys at T1 and T3 at an acceptable length, snack intake was only assessed at T2.

At T3, participants answered the same questionnaires as in the initial survey. This was part of a student project and in order for it to be kept at an acceptable length, some strategies as well as the flexible and rigid restraint measures^(19)^ were removed. This follow-up was after the COVID-19 lockdowns; hence, questions about change in eating behaviour and working/living conditions due to lockdown were added but were not used in these analyses.

### Analysis

All analyses were performed in IBM SPSS statistics 28.0.1.1. For the moderation analysis, the PROCESS macro for SPSS was used^(20)^. For calculations and a description of covariates, see supplemental data C.

#### Factor analysis of the strategies

As the number of individual strategies was considerable (*n* = 41), to limit the risk of multicollinearity and reduce the number of predictors in our models, the frequency of strategy use was used to reduce the number of strategies using principal axis factoring with promax rotation. With the help of a scree plot the number of factors was identified, and another principal axis factoring was conducted only with the identified factors. Only items with factor loading > 0·3 were included, and a mean of each factor per participant was calculated. As participants only had to answer the question about subjective effectiveness if they used the respective strategy, the number of answers for some strategies were extremely low. These low numbers of answers were not sufficient to conduct principal axis factoring for subjective effectiveness. Therefore, the same strategies which grouped into the factors of frequency of use were used for the subjective effectiveness factors. These factors were used in the following analyses.

#### Relationship between BMI and strategies

To address the first hypothesis, the relationship between the frequency and subjective effectiveness of the strategies and BMI were evaluated using a multiple regression with bootstrapping (BCa, 1000 repetitions). Hierarchical entry was used with the covariates (age, gender, living conditions, meal style and physical activity) in the first and the frequency and effectiveness factors in the second step. The test was changed from the pre-registered unrelated *t* tests to multiple regression to retain power by keeping BMI as a continuous variable.

#### Moderation of the change in BMI by strategy

To address the second hypothesis, we identified factors that influenced change in BMI from T1 (predictor) to T2 (outcome). Moderation analyses were conducted with the frequency and subjective effectiveness of the strategies as moderators. The following variables were used as covariates: ethnicity, currently dieting, age, diet score, cognitive and rigid restraint, emotional, external eating, and craving. Moderation analyses with BMI at T1 and T2 as predictor and outcome variables, respectively, were chosen instead of multiple regression with change scores (as mentioned in the pre-registration) to avoid a loss of power.

#### Self-reported intake of healthy and unhealthy snacks

For the third hypothesis, two simultaneous multiple regression analyses were conducted to investigate if the frequency and subjective effectiveness of strategies predicted (healthy and unhealthy) snack intake. As covariates, age, gender and ethnicity were considered.

#### Exploratory analysis

To explore the change in BMI over time, a Friedman test with Bonferroni correction was performed. Additionally, the influence of the frequency of use and subjective effectiveness of strategies on change in BMI between the three time points were assessed with multivariate regression with frequency of use and subjective effectiveness as predictors and change in BMI between each time point as outcome variables. These changes in BMI scores were calculated by subtracting the later BMI from the earlier one, for example, BMI T3 – BMI T2. Therefore, a higher change score means an increase in BMI and weight.

## Results

### Participant characteristics for baseline (T1)

Most participants were female (76·90 %), White (78·26 %) and non-smokers (92·40 %), with an average age of 34·41 ± 13·61 years. Most participants ate meat (79·89 %) and did about 3·5 h per week of light physical activity. Further participants’ characteristics can be found in Table 1.

**Table 1:** Participants’ characteristics

Variable	Baseline/T1 (*n* = 368)	
Gender†		
Male	83	22·60
Female	283	76·90
Neutral	2	0·50
Gender identity (*n*, % yes)	367	99·70
Ethnicity†		
White	288	78·26
Mixed, multiple ethnic groups	8	2·17
Asian, Asian British	62	16·85
Black, African, Caribbean, Black British	3	0·82
Not listed ethnic group	6	1·63
Prefer not to say	1	0·27
Occupation†		
Student	155	42·12
Full-time employed	123	33·42
Part-time employed	42	11·41
Self-employed	20	5·43
Parent, carer, unemployed	27	7·34
Missing	1	0·27
Living condition†		
Alone	48	13·04
Shared flat	102	27·72
With partner	99	26·90
With family	118	32·07
Missing	1	0·27
Smoking (*n*, % no)	340	92·40
Drinking alcohol (*n*, % no)	100	27·20
Currently dieting (*n*, % no)	283	76·90
BMI T1 (kg/m^2^)‡	25·06	5·04
Underweight†	6	1·63
Healthy’ weight†	208	56·52
Overweight†	90	24·46
Obesity†	56	15·22
Missing†	8	2·17
Diet score‡	0·84	1·03
Never†	168	45·65
1–3 times in last 3 years†	133	36·14
4–6 times in last 3 years†	35	9·51
7–10 times in last 3 years†	13	3·53
11 times or more†	16	4·35
Missing†	3	0·82
Bariatric surgery (*n*, % no)	367	99·70
Meal style†		
Vegetarian	30	8·15
Vegan	21	5·71
Pescetarian*	22	5·98
Omnivor	294	79·89
Missing	1	0·27
Age (years)‡	34·41	13·61
Weight (kg)‡	70·31	16·73
Highest weight (kg)‡	76·00	19·15
Height (cm)‡	166·98	9·02
Weight suppression (kg)‡	5·72	6·81
Weight suppression (%)‡	8·14	9·64
Physical activity (min/week)‡		
Light	211·49	191·86
Moderate	38·71	80·30
Vigorous	61·81	106·85
Strength	63·20	100·09
Mean physical activity score‡	67·93	64·34

* Eating no meat but fish.

† *n* (%).

‡ M (sd).

### Participant characteristics for follow-ups (T2 and T3)

Most participants were female (T2: 86·50 %, T3: 82·40 %) and had a healthy to overweight BMI (T2: BMI T1 = 25·19 ± 5·28 kg/m^2^; T3: BMI T1 = 25·71 ± 5·71 kg/m^2^). Table 2 gives a full overview of the characteristics of the participants who participated in the follow-ups compared with participants who did not.

**Table 2:** Participant characteristics of the responders and non-responders of the follow-ups

Variable	Follow-up 1/T2				*P*-value§	Follow-up 2/T3				*P*-value§
	Responder (*n* = 170)		Non-responder (*n* = 198)			Responder (*n* = 51)		Non-responder (*n* = 317)		
Gender†					< 0·001**					0·137
Male	23	13·50	60	30·30		8	15·70	75	26·70	
Female	147	86·50	136	68·70		42	82·40	241	76·00	
Not stated	0	0·00	2	1·00		1	2·00	1	0·30	
Gender identity (*n*, % yes)	170	100·00	197	99·50	–	50	98·00	317	100·00	–
Ethnicity†					< 0·001**					0·128
White	151	88·80	137	69·20		48	94·10	240	75·70	
Mixed, multiple ethnic groups	5	2·90	3	1·50		1	2·00	7	2·20	
Asian, Asian British	10	5·90	52	26·30		2	3·90	60	18·90	
Black, African, Caribbean, Black British	1	0·60	2	1·00		0	0·00	3	0·90	
Not listed ethnic group	3	1·80	3	1·50		0	0·00	6	1·90	
Prefer not to say	0	0·00	1	0·50		0	0·00	1	0·30	
Occupation†					0·079					0·003*
Student	62	36·50	93	47·00		15	29·41	140	44·20	
Full-time employed	62	36·50	61	30·80		15	29·40	108	34·10	
Part-time employed	24	14·10	18	9·10		14	27·50	28	8·80	
Self-employed	6	3·50	14	7·10		2	3·90	18	5·70	
Parent, carer, unemployed	15	8·80	12	6·10		5	9·80	22	6·90	
Missing	1	0·60	0	0·00		0	0·00	1	0·30	
Living condition†					0·103					0·279
Alone	24	14·10	24	12·10		10	19·61	38	12·00	
Shared flat	40	23·50	62	31·30		11	21·60	91	28·70	
With partner	55	32·40	44	22·20		11	21·60	88	27·80	
With family	51	30·00	67	33·80		19	37·30	99	31·20	
Missing	0	0·00	1	0·50		0	0·00	1	0·30	
Smoking (*n*, % no)	159	93·50	181	91·40	0·555	48	94·10	292	92·10	0·781
Drinking alcohol (*n*, % no)	41	24·10	59	29·80	0·241	15	29·40	85	26·80	0·735
Currently dieting (*n*, % no)	129	75·90	154	77·80	0·174	40	78·40	243	76·70	0·859
Diet score T1‡	0·91	1·09	0·78	0·99	0·710	0·96	1·23	0·82	1·00	0·695
Never†	71	41·80	97	49·00	–	22	43·14	146	46·10	–
1–3 times in last 3 years†	67	39·40	66	33·30		21	41·20	112	35·30	
4–6 times in last 3 years†	14	8·20	21	10·60		1	2·00	343	10·70	
7–10 times in last 3 years†	6	3·50	7	3·50		2	3·90	11	3·50	
11 times or more†	10	5·90	6	3·00		5	9·80	11	3·50	
Missing†	2	1·20	1	0·50		0	0·00	3	0·90	
Number of diets‡	1·58	1·00	1·53	0·87	–	1·69	1·20	1·53	0·88	–
Bariatric surgery (*n*, % no)	170	100·00	197	99·50	1·000	51	100·00	316	99·70	1·000
Future weight plans†					–					–
I plan to lose weight in the next 30 d	45	26·50	34	17·20		11	21·60	68	21·50	
I plan to lose weight in the next 6 months	60	35·30	71	35·90		19	37·30	112	35·30	
I plan to increase my weight in the next 6 months	1	0·60	5	2·50		0	0·00	6	1·90	
I plan to increase my weight in the next 30 d	3	1·80	3	1·50		0	0·00	6	1·90	
I plan to maintain my weight	56	32·90	71	35·90		20	39·20	107	33·80	
Missing	5	2·90	14	7·10		1	2·00	18	5·70	
Meal style†					0·062					0·538
Vegetarian	19	11·20	11	5·60		4	7·80	26	8·20	
Vegan	10	5·90	11	5·60		5	9·80	16	5·00	
Pescetarian	14	8·20	8	4·00		2	3·90	20	6·30	
Omnivor	127	74·70	167	84·30		40	78·40	254	80·10	
Missing	0	0·00	1	0·50		0	0·00	1	0·30	
Age T1 (years)‡	36·76	13·80	32·32	13·13	< 0·001**	41·14	14·98	33·30	13·07	< 0·001**
BMI T1 (kg/m^2^)‡	25·19	5·28	24·93	4·84	0·694	25·71	5·71	24·95	4·93	0·414
Underweight†	3	1·80	3	1·50	0·759||	2	3·90	4	1·30	0·150||
Healthy’ weight†	92	54·10	116	58·60		22	43·10	186	58·70	
Overweight†	45	26·50	45	22·70		15	29·40	75	23·70	
Obesity†	28	16·50	28	14·10		10	19·60	46	14·50	
Missing†	2	1·20	6	3·00		2	3·90	6	1·90	
BMI T2 (kg/m^2^)‡	25·51	5·44	–	–	n/a	26·48	5·97	25·22	5·26	–
BMI T3 (kg/m^2^)‡	27·06	5·87	25·18	6·75	–	26·64	6·05	–	–	n/a
Weight suppression (kg)‡	6·23	6·64	5·27	6·95	0·065	6·62	8·12	5·57	6·58	0·520
Weight suppression (%)‡	8·87	9·30	7·50	9·92	0·037*	9·06	10·95	0·08	0·09	0·600
Physical activity (min/week)‡										
Light	234·83	214·15	190·27	166·85	–	202·06	185·57	213·07	193·15	–
Moderate	38·85	84·73	38·59	76·79	–	41·79	76·33	38·24	81·02	–
Vigorous	69·77	129·14	54·97	82·89	–	58·25	81·98	62·39	110·47	–
Strength	64·65	89·42	61·96	108·68	–	49·67	66·92	65·30	104·23	–
Mean physical activity score‡	71·09	70·64	65·41	58·92	0·788	62·51	65·06	68·79	64·32	0·329
Sum of frequency of intake T2‡										
Healthy snacks	32·06	9·86	–	–	n/a	32·08	10·47	32·05	9·72	–
Unhealthy snacks	23·33	6·85	–	–	n/a	23·21	5·49	23·37	7·23	–
Mean frequency of intake T2‡										
Healthy snacks	2·91	0·90	–	–	n/a	2·92	0·95	2·91	0·88	–
Unhealthy snacks	2·12	0·62	–	–	n/a	2·11	0·50	2·12	0·66	–

* A significant difference at *p* < 0·05 level between the two groups using Mann–Whitney (continuous variables) and χ^2^ test (categorical variables).

** A significant *p* < 0·001 level between the two groups using Mann–Whitney (continuous variables) and *χ* ^2^ test (categorical variables).

† *n* (%).

‡ M (sd).

§ Comparison of responder to non-responder.

|| Comparison across BMI categories between responders and non-responders.

### Factor analysis of frequency of use and subjective effectiveness of strategies (initial questionnaire)

The factor analysis grouped the strategies into two factors based on the frequency of use: (1) diet, exercise, reduction of temptations and cognitive strategies (Table 3); and (2) planning, preparation and eating style (Table 4). Ten strategies did not load to any factor (supplemental data C) and were therefore excluded from further analyses. The mean scores of the frequency of use factors were significantly correlated (r_S_ = 0·28, *P* < 0·001).

**Table 3:** Strategies, which group together in factor 1 ‘Planning, preparation and eating style’ with their factor loadings, mean frequency of use and mean effectiveness with sd

Factor 1 = Planning, preparation and eating style	Factor loading	Mean frequency per strategy	sd	Mean effectiveness per strategy	sd
Taking time for cooking	0·780	3·81	1·06	69·45	25·22
Varying ones diet	0·637	3·03	0·98	46·44	26·80
Plan meals and snacks in advance	0·594	3·18	1·26	69·18	24·57
Trying new and different dishes	0·562	3·29	0·95	41·67	26·88
Bring own food to uni or work	0·546	3·47	1·35	70·98	25·69
Bigger meals less often	0·531	3·70	1·08	67·37	24·97
Pay attention what you buy	0·524	3·61	1·03	74·43	19·84
Replace unhealthy with healthier choices	0·494	3·10	1·06	69·64	20·90
Consistent eating times	0·430	3·40	1·56	59·72	26·37
Eating vegetarian, vegan or pescetarian	0·397	3·07	1·29	46·36	30·07
Following flexible approach	0·345	3·33	1·03	49·30	28·26
Cooking meals in advance	0·341	2·37	1·20	62·56	25·23
Success as incentive to continue	0·315	3·00	1·05	64·36	22·15
Feeling if oneself is full or good after meal	0·309	3·53	1·04	65·26	23·80
Mean per factor (sd)		3·28	0·59	60·57	16·02

**Table 4:** Strategies, which group together in factor 2 ‘Diet, exercise, reduction of temptations, and cognitive strategies’ with their factor loadings, mean frequency of use and mean effectiveness with sd

Factor 2 = Diet, exercise, reduction of temptation and cognitive strategies	Factor loading	Mean frequency per strategy	sd	Mean effectiveness per strategy	sd
Distraction from thoughts of temptation	0·637	2·58	1·11	57·59	24·15
Setting yourself rules	0·599	2·72	1·22	61·55	24·23
Avoiding tempting situations	0·552	3·00	0·98	61·05	22·60
Using a commercial diet	0·483	1·50	0·91	56·03	25·42
Fasting for a specific amount of time	0·474	1·60	0·89	56·68	28·01
Giving oneself specific time for indulgences	0·456	2·68	1·25	51·72	26·21
Buying expensive foods	0·452	1·85	1·02	42·89	26·11
Negative feedback as motivation	0·445	2·46	1·29	60·09	25·91
Filling up of portions with low-caloric options	0·416	2·89	1·14	65·69	23·34
Postponing of intake	0·411	2·74	1·13	52·37	24·87
Using apps	0·402	2·05	1·32	56·92	29·80
Storing in places which are hard to reach	0·399	2·11	1·20	60·33	24·96
Not having temptations around	0·371	3·41	1·17	79·43	18·65
Seeing tempting foods as reward/treat	0·350	3·03	1·20	39·11	28·64
Eating smaller meals more often	0·340	2·12	1·09	54·78	23·20
Exercise to regulate hunger and temptation	0·322	2·88	1·03	0·00	0·00
Exercise to balance calorie intake	0·316	2·44	1·01	47·77	27·82
Mean per factor (sd)		2·47	0·55	51·95	11·98

### Relationship between BMI and strategies at T1

The overall model for predicting BMI was significant (F-change(4,248) = 4·54, R^2^-change = 0·06, *P* = 0·001). The more frequent use of factor 2 ‘Planning, preparation and eating style’ strategies was related to a lower BMI (t = –1·99, *β* = –0·15, *P* = 0·048), while a higher frequency of use of the factor 1 ‘Diet, exercise, reduction of temptations, and cognitive strategies’ was predictive of a higher BMI (t = 3·60, *β* = 0·24, *P* < 0·001). The subjective effectiveness of both factors were not related to BMI (*P* > 0·363).

### Moderation of the change in BMI by strategies

For frequency of use, neither of the strategy factors moderated change from BMI T1 to T2 (factor 1 ‘Diet, exercise, reduction of temptations, and cognitive strategies’: *b* = 0·02, se = 0·03, *t* = 0·70, *P* = 0·487, 95 % CI = –0·044, 0·093; factor 2 ‘Planning, preparation and eating style’: *b* = 0·03, se = 0·03, *t* = 1·05, *P* = 0·296, 95 % CI = –0·025, 0·082).

Similarly, the change from BMI T1 to T2 was not moderated by either of the subjective effectiveness factors (factor 1 ‘Diet, exercise, reduction of temptations, and cognitive strategies’: *b* = 0·00, se = 0·00, *t* = 0·23, *P* = 0·816, 95 % CI = –0·002, 0·003; factor 2 ‘Planning, preparation and eating style’: *b* = –0·00, se = 0·00, *t* = –0·25, *P* = 0·800, 95 % CI = –0·003, 0·002).

### Self-reported intake of healthy and unhealthy snacks

For healthy snacks, neither frequency of use nor subjective effectiveness of strategies at T1 predicted the self-reported intake (frequency) at T2 (*P* > 0·116).

For unhealthy snacks, the overall change model was significant (F-change(4,154) = 5·20, R^2^-change = 0·11, *P* < 0·001). Post hoc revealed only a significant effect for the subjective effectiveness of factor 2 ‘Planning, preparation and eating style’ at T1 which decreased self-reported intake at T2 (t = –2·20, *β* = –0·01, *P* = 0·029).

### Exploratory analyses – change of BMI over time and influence of strategies

There was a significant difference in BMI between time points (*n* 38, χ^2^(2,38) = 11·49, *P* = 0·003). The change from T1 to T2 and T3 were both significant with an increase in BMI over time (BMI T1 = 26·08 ± 5·71, T2 = 26·65 ± 5·96, T3 = 27·11 ± 5·94; Bonferroni-corrected: T1 *v*. T2: *P* = 0·048; T1 *v*. T3: *P* = 0·006), but not between the follow-ups (adj. *P* = 1).

When the influence of the frequency of use and subjective effectiveness of factors on the change in BMI over time was considered, only the subjective effectiveness of strategies around ‘Diet, exercise, reduction of temptations, and cognitive strategies’ (factor 1) was significant (F(2,32) = 4·56, *P* = 0·018). It only affected the change in BMI T1 to T3 (F(1,33) = 5·41, adj. R^2^ = 0·15, *β* = 0·09, *P* = 0·026) and T2 to T3 (F(1,33) = 8·64, adj. R^2^ = 0·21, *β* = 0·10, *P* = 0·006) positively. Therefore, the more effective the strategy was perceived, the stronger the increase in BMI.

## Discussion

This study aimed to assess the frequency of use and subjective effectiveness of self-directed strategies to manage intake of tempting foods and how these predict (change in) BMI and snack intake. Two strategy factors were quantitatively identified: (1) diet, exercise, reduction of temptations, and cognitive strategies, and (2) planning, preparation, and eating style.

A higher frequency of use of the strategies around ‘planning, preparation, and eating style’ (factor 2) was related to a lower BMI at T1. However, this did not predict a change in BMI at T2 and was not associated with healthy or unhealthy snack intake. Subjective effectiveness was not correlated to BMI, change in BMI or healthy snack intake, but a higher subjective effectiveness predicted lower unhealthy snack consumption. This indicates that people might use this strategy for maintenance of weight loss and lifetime weight, as well as eating fewer unhealthy snacks. BMI was maintained over the follow-up period across all BMI groups (underweight, ‘healthy’ weight, overweight and obesity) which suggests that the strategies are more effective for weight (loss) maintenance than for prospective weight loss. Kruseman *et al.* ^(21)^ found that strategies to manage intake are not only limited to weight loss maintenance but also to maintain the lifetime weight in lean individuals, which is supported by our findings. Also, studies looking at strategies for weight loss maintenance to keep a healthy diet in participants with a healthy weight and to reduce tempting food intake separately found similar approaches^(7,9,22)^. Interestingly, only the subjective effectiveness but not the frequency of use of strategies was related to less unhealthy snack consumption. One explanation might be that our study only assessed usage and subjective effectiveness of strategies as well as snack intake cross-sectionally, not longitudinally. Therefore, no conclusion about the direction of effect can be made. Rather than the subjective effectiveness led to a lower snack intake, participants attributed a low intake of unhealthy snacks to the strategies they are using and described them as effective.

The findings of this study present an opportunity to manage BMI by emphasising the importance of planning and cooking of meals when giving advice for weight maintenance and a healthier diet. Preparation and planning of home-cooked meals and snacks, and eating style (e.g. replacing unhealthy with healthy food choices, taking time for cooking and trying new dishes) were used frequently in the individuals’ day-to-day life and hence seem to be acceptable. The current as well as previous research found these strategies are related to lower BMI, maintaining a stable weight, and even weight loss and a lower likelihood for having overweight^(23–30)^. This might be due to better cooking skills and planning which lead to healthier eating and lower BMI^(26)^. However, cooking and cooking skills are low especially in men^(31)^ and individuals with low income^(27)^ independently of BMI. Therefore, one way for healthier eating and BMI might be to provide environments which facilitate learning how to plan and prepare meals, for example, in school as learning cooking in younger age improves cooking skills and healthier eating in later life^(31)^. As time, costs and skills are main barriers for home cooking^(28)^, a focus should be on preparation of easy and quick dishes with affordable and healthy ingredients. Planning meals might support thinking about quick and cheap recipes, buying the needed ingredients in advance, and therefore increase the likelihood of cooking at home. Interventional studies indicate that cooking and education classes improve healthy eating^(32)^ and reduce snacking because of the anticipation of a full meal for dinner and switching unhealthy for healthy snacks^(29)^. To maintain home cooking and meal planning, social media and digital technology such as tutorials and apps could be used. It is important to fit food planning and preparation into the daily schedule as more frequent eating of home-cooked meals is strongly associated with healthier eating^(25)^.

In contrast to factor 2 strategies, factor 1 strategies relating to ‘diet, exercise, reduction of temptations, and cognitive strategie’ were used more frequently by people with higher BMI. One possibility is that individuals with a higher BMI might be more likely to engage in weight loss attempts than individuals with a lower BMI^(33)^ and therefore use commercial diets, apps and exercise more often. Also, avoidance of tempting situations or foods and cognitive strategies are commonly used for weight loss^(3)^. However, there lacked a concomitant relationship of BMI with both the subjective effectiveness of these strategies and the objective effectiveness represented by no association between frequency of use and subjective effectiveness with change in BMI from T1 to T2 as well as intake of snacks. The relative disconnect between frequency of use of strategies by individuals with overweight and subjective/objective effectiveness is in line with Hartmann-Boyce *et al.* ^(3)^. They state that some strategies which are used for self-directed weight loss are not recommended by self-help weight loss interventions. This includes strategies such as scheduling of physical activity and weight management aids, which are mentioned in their review of qualitative studies. However, when compared with a systematic review^(34)^, only six and two studies out of thirty-nine recommended scheduling and aids, respectively^(3)^. This discrepancy between recommended and used strategies should be considered by clinical practitioners and self-help websites/apps when giving weight loss or weight loss maintenance advice.

Living healthily and managing weight is difficult in our current food environment^(35)^. One reason might be that tempting foods in our environment trigger reward processes and attentional bias which are enhanced and longer-lasting for individuals who struggle managing weight^(36)^. As strategies such as reduction of temptations (e.g. avoiding tempting situations) and cognitive strategies (e.g. distraction from tempting thoughts) need constant effort, a high amount of self-control and executive function, this can lead to mental fatigue and lapses in the effort to resist temptations^(37)^. This might raise the concern that these strategies work on a smaller scale at home or at work where there are less triggers but are likely not enough in our current food environment where tempting foods are more omnipresent. Therefore, efforts by the individual will not be enough to reduce the global obesity epidemic sufficiently. Instead changes in the food environment implemented by a combined effort of urban planners, policymakers, food industry and researchers are needed (for a critical commentary about shifting the responsibility from the individual to policymakers, see ref. 35).

When looking at BMI over time, BMI increased significantly from baseline to the first follow-up (3 months, T2) but then stabilised to the second follow-up after 3 years (T3). Between the two follow-ups, the COVID-19 pandemic started at the end of 2019, which led to an increase in BMI^(38)^ because physical activity decreased, while sedentary behaviour, snacking frequency, food consumption and emotional eating increased^(39,40)^. However, the present study did not support these findings which might be due to different reasons. By the time of the second follow-up, the lockdowns eased and people had the chance to join sport clubs and increase the physical activity (for adolescents: see ref. 41), which could support stabilising the BMI^(42)^. However, not many studies looked at the changes in weight, eating behaviour and physical activity 1·5 years after lockdown. Also, the sample size for this analysis was low (*n* = 38) and might not have enough power (for a power calculation, see supplemental data E).

Some limitations must be considered. First, all measurements were self-reported. Even though weight and height might be under- or overreported, BMI categorisation was shown to still be reliable^(43)^. Second, the first follow–up period was only 3 months and the second did not assess the frequency of use and effectiveness of all strategies as well as snack intake, and therefore long-term effects cannot be identified. In future research, a longer follow-up period measuring all variables should be used. Third, subjective effectiveness of a strategy was only reported by participants who indicated that they use that strategy. This limited the sample size of some strategies strongly. To increase the power, individuals not using a strategy could indicate the possible effectiveness if they would use it. Another possibility is to have a bigger sample size leading to a reasonable number also for analyses of subjective effectiveness. Fourth, with the sample size of 368 participants at T1 and 170 at T2, small-to-medium and medium-to-large effect sizes, respectively, can be picked up in the moderation analyses^(44)^. However, in psychology most effects are small and could therefore not been found^(45–47)^. To increase power and to also discover effects of small size, a bigger sample size is needed. Fifth, neither adherence nor the exclusion of strategies in the follow-ups were measured. This information would help to understand the change in BMI and the influence of strategies on snack intake further. Lastly, some of the strategies might be used to reduce overall food intake (e.g. using a commercial diet, apps or exercise), and/or to eat healthier (e.g. cooking at home) instead of specifically reducing the consumption of tempting foods. Having separate questionnaires for each of the purposes could give a clearer picture of the objective effectiveness of the strategies to eat healthier, lose or maintain weight.

‘Tempting foods’ was not defined by the researchers as every person might find different foods tempting. However, particularly in ‘Western’ cultures, tempting foods are often assumed to be foods which we should eat less of such as ultra-processed foods and following a Western diet. This does not necessarily be the case. Therefore, future research could try to better understand how people define ‘tempting foods’ (foods which intake should be reduced *vs.* possibly even increased) and if there are cultural differences.

Taken together, strategies around ‘diet, exercise, reductions of temptations, and cognitive strategies’ were used more frequently by individuals with higher BMI possibly for weight loss but are not perceived as more effective and do not influence BMI change and snack intake. Contrary, strategies around ‘planning, preparation, and eating style’ are more frequently used by participants with lower BMI but perceived as similarly effective, while being related to a stable weight; thus, they are more effective for weight maintenance.

## Supporting information

Gatzemeier et al. supplementary material Gatzemeier et al. supplementary material

## Supplementary material

For supplementary material accompanying this paper visit https://doi.org/10.1017/S1368980024000697
