# Supplementary material for: Self-identified strategies to manage intake of tempting foods: cross-sectional and prospective associations with BMI and snack intake
Source: Public Health Nutr. 2024 Mar 20;27(1):e107. doi: 10.1017/S1368980024000697 (PMC11036431; doi:10.1017/S1368980024000697)
Supplement: Gatzemeier et al. supplementary material [file S1368980024000697sup001.docx]

# Supplemental data

## Power calculations for each of the analysis

G*Power 3.1 (1) was used for all a-priori sample size calculations with alpha = .05, power = 0.8, and a Cohen’s f = 0.18 or f^2^ = 0.08 to detect a small-to-medium effect size (based on e.g., 2). It should be noted that these analyses were run after the data collection ended.

For the assessment of the influence of the frequency of use and perceived effectiveness on BMI T1, a multiple regression (R^2^ increase) was used. For an f^2^ = 0.08, number of tested predictors 4 (2 factors for frequency of use and 2 factors of perceived effectiveness) and with a total number of predictors of 9 (additional 5 covariates), a sample size of 155 participants was needed.

To assess the effect of frequency of use and perceived effectiveness on healthy and unhealthy snack intake, two separate multiple regressions were run. For an f^2^ = 0.08, number of tested predictors 4 (2 factors for frequency of use and 2 factors of perceived effectiveness) and number of total predictors 7 (additional 3 covariates), a sample size of 155 participants was required.

Friedman test was run to test the change of BMI over the three timepoints. As G*Power does not have an option for Friedman test, a repeated measure, within subject ANOVA was chosen. If f = 0.18, number of groups = 1, number of measurements = 3, a nonsphericity correction = 0.5 (1) and correlation among repeated measures = .5, then a sample size of 83 participants is needed.

A multivariate regression was used to assess the influence of the frequency of use and subjective effectiveness of the strategies on the change in BMI. Sample size calculation for one outcome variable is conducted with multiple regression (R^2^ increase) selected. The sample size for each outcome variable (difference in BMI between T1 and 2, between T1 and 3, and between T2 and 3) is the same. For an f^2^ = 0.08, number of tested predictors 4 (2 factors for frequency of use and 2 factors of perceived effectiveness) and number of total predictors 4 (no additional covariates), a sample size of 155 participants was required.

## List of psychological and eating behaviour traits

A range of psychological and eating behaviour traits were assessed to identify if individuals with particular traits use strategies to manage intake of tempting foods more or less frequent. Following questionnaires were administered to collect the data about traits:

- Dutch Eating Behaviour Questionnaire (DEBQ; 3) to assess external, emotional, and restrained eating
- Flexible and Rigid Control sub-scales (4) to assess flexible and rigid restraint
- Barratt Impulsiveness Scale (BIS-11; 5) to assess motor, attentional, and non-planning impulsiveness
- Zimbardo Time Perspective Inventory (ZTPI; 6) to assess past positive, past negative, present hedonistic, present fatalistic, and future time perspective
- Reduced Food Craving Questionnaire – Trait (FCQ-T-r; 7) to assess food craving

## Calculations of covariates

Some variables were included in the analyses as possible covariates. In the following the calculations of these variables are explained.

- BMI:
  The common equation was used: BMI=weight/height^2^
- Weight suppression:
  Weight suppression is the difference between the highest weight since current height and current weight in kg and % of current weight
- Physical Aciticity Score:
  In the protocol pre-registered with the OSF, the intention was to use the Godin and Shephard Leisure-Time Physical Activity Questionnaire (Godin, 2011) to assess physical activity. The questions were changed to measure how many minutes per week participants take part in light, moderate, vigorous activities and strength workout due to the conventions in the department and a more common way of thinking in amount of time spent on physical activities. Therefore, it was not possible to calculate the score as originally mentioned in the pre-registration. Instead a Physical Activity Score (PAS) was created:

PAS= (1*light activity+2*medium activity+3*vigorous activity+2*strength workout)/8

- History of Dieting/diet score:
  A diet score comparable to Price, Higgs and Lee (8) was created by combining the information provided by participants about their dieting history and current dieting status. Never dieted was coded with 0, 1-3 time with 1, 4-6 times with 2, 7-10 times with 3 and more than 11 times with 4.

## Strategies which were not included in the factors

Strategies with a factor loading <.3 were not included in the factors. These strategies are presented here:

| Factor loading <.3 | Mean frequency per strategy (SD) | Mean effectiveness per strategy (SD) |
| --- | --- | --- |
| Choosing food depending on time of day | 1.81 (1.13) | 56.93 (24.56) |
| Reflect on reasons for eating | 2.49 (1.15) | 52.42 (25.86) |
| Taking time during meal | 2.37 (1.12) | 51.16 (25.69) |
| Buying smaller amounts | 2.29 (1.23) | 55.35 (24.02) |
| Grocery shopping online | 1.97 (1.24) | 57.78 (30.01) |
| Shopping for whole week | 3.09 (1.30) | 63.76 (25.52) |
| Seeing food as necessity | 3.35 (1.29) | 46.65 (28.89) |
| Taking smaller portions | 2.92 (1.10) | 60.58 (23.74) |
| Specific routine after meal | 2.44 (1.31) | 45.17 (27.68) |
| Seeing foods as "fuel" | 2.33 (1.21) | 53.37 (24.89) |
| Extraction Method: Principal Axis Factoring.   Rotation Method: Promax with Kaiser Normalization. | | |

1. Post-hoc power analysis

Sample size for the analysis looking at the change in BMI over the three timepoints was quite low (*n* = 38). To test if the power was high enough, post-hoc power analysis with G*Power for a repeated measure, within factors ANOVA with effect size f=0.18, α=0.05, N=38, number of groups 1, number of measurements 3 (T1, T2, T3), correlation among repeated measures of 0.5, and a nonsphericity correction of 0.5 (9, 10) was run. It revealed a power of 0.46. The information for this power analysis were based on a repeated measure ANOVA with Greenhouse-Geisser correction for nonsphericity instead of the Friedman test as the Friedman test does not give the necessary information.

## Literature

1. Faul F, Erdfelder E, Lang AG, Buchner A. G*Power 3: a flexible statistical power analysis program for the social, behavioral, and biomedical sciences. Behav Res Methods. 2007;39(2):175-91.

2. Schippers M, Adam PCG, Smolenski DJ, Wong HTH, de Wit JBF. A meta-analysis of overall effects of weight loss interventions delivered via mobile phones and effect size differences according to delivery mode, personal contact, and intervention intensity and duration. Obesity Reviews. 2017;18(4):450-9.

3. Van Strien T, Frijters JER, Bergers GPA, Defares PB. The Dutch Eating Behavior Questionnaire (DEBQ) for Assessment of Restrained Emotional, and External Eating Behavior. International Journal of Eating Disorders. 1986;5(2):295-315.

4. Westenhoefer J, Stunkard AJ, Pudel V. Validation of the flexible and rigid control dimensions of dietary restraint. Int J Eat Disord. 1999;26(1):53-64.

5. Patton JH, Stanford MS, Barratt ES. Factor structure of the Barratt impulsiveness scale. J Clin Psychol. 1995;51(6):768-74.

6. Zimbardo P, Boyd JN. Putting time in perspective: A valid, reliable individual-differences metric. Journal of Personality and Social Psychology. 1999;77(6):1271-88.

7. Meule A, Hermann T, Kubler A. A short version of the Food Cravings Questionnaire-Trait: the FCQ-T-reduced. Front Psychol. 2014;5:190.

8. Price M, Higgs S, Lee M. Self-reported eating traits: Underlying components of food responsivity and dietary restriction are positively related to BMI. Appetite. 2015;95:203-10.

9. Faul F, Erdfelder E, Buchner A, Lang AG. Statistical power analyses using G*Power 3.1: tests for correlation and regression analyses. Behav Res Methods. 2009;41(4):1149-60.

10. Celis-Morales C, Livingstone KM, Woolhead C, Forster H, O’Donovan CB, Macready AL, et al. How reliable is internet-based self-reported identity, socio-demographic and obesity measures in European adults? Genes & Nutrition. 2015;10(5):28.
